# Supplementary material for: Vesicle shape transformations driven by confined active filaments
Source: Nat Commun. 2021 Dec 13;12:7247. doi: 10.1038/s41467-021-27310-8 (PMC8668962; doi:10.1038/s41467-021-27310-8)
Supplement: Supplementary file 1 — Supplementary Information [file 41467_2021_27310_MOESM1_ESM.pdf]

Vesicle shape transformations driven by confined active filaments

Matthew S. E. Peterson,[1] Aparna Baskaran,[1, *] and Michael F. Hagan[1, †]
[1] *Martin A. Fisher School of Physics, Brandeis University, Waltham, MA, 02453*
(Dated: November 11, 2021)

SUPPLEMENTARY NOTES

1. Filament persistence time

In this section we compute the persistence length of the center of mass motion of the active filaments, $l_{\mathrm{p}}^{\mathrm{COM}}$, to determine the regions in parameter space that correspond to the strong confinement limit. Under strong confinement, almost all rods are on the vesicle surface at all times, while below this limit rods are found throughout the vesicle interior. Strong confinement occurs when the filament motion persistence length is larger than the vesicle size, i.e., $l_{\mathrm{p}}^{\mathrm{COM}} \gtrsim 2R_{\mathrm{ves}}$ [1, 2]. This limit can be understood by noting that when a self-propelled filament collides with the vesicle surface it is reoriented into the surface tension plane. When $l_{\mathrm{p}}^{\mathrm{COM}} \gtrsim 2R_{\mathrm{ves}}$, rotation of the filament away from the surface occurs more slowly than realignment with the surface due to subsequent collisions.

The autocorrelation timescale for rotation of a rigid rod is $\tau_{\mathrm{corr}} = m\gamma\sigma^2 a^3/12k_{\mathrm{B}}T \propto a^3$ [3], which is consistent with simulations of isolated filaments, both with and without activity (see Supplementary Figure 1). The persistence length of motion $l_{\mathrm{p}}^{\mathrm{COM}}$ is therefore

$$l_{\mathrm{p}}^{\mathrm{COM}} = v_0\tau_{\mathrm{corr}} = \frac{\sigma^2 a^3 f_{\mathrm{a}}}{12k_{\mathrm{B}}T} = \frac{1}{12}\sigma a^3 \mathrm{Pe}, \tag{1}$$

where we have used $v_0 = f_{\mathrm{a}}/m\gamma$ as the active propulsion velocity. Recalling that the system enters the strong confinement limit when this persistent length is on the order of the vesicle diameter, $2R_{\mathrm{ves}}$, the threshold aspect ratio for strong confinement is

$$a_{\mathrm{SC}} \gtrsim \left(\frac{24R_{\mathrm{ves}}}{\sigma\mathrm{Pe}}\right)^{1/3}. \tag{2}$$

For the parameters of Fig. 1 in the main text ($\mathrm{Pe} = 8$ and $R_{\mathrm{ves}} = 25\sigma$), the threshold aspect ratio is $a_{\mathrm{SC}} \approx 4.2$.

2. Competition between rotation and collision timescales

a. Rotation timescale: Consider a rigid, self-propelled rod, consisting of $N+1$ monomers of mass m and bond length b, that is moving toward a flat boundary. The rod is propelled by an active force with magnitude f_{a} along the rod's tangent $\hat{\mathbf{p}}$. At the point of collision with the boundary, the rod will begin to rotate parallel to the wall. The rotation will happen over a timescale equal to the that over which the rod would have moved its own length, $\tau_{\mathrm{rot}} \sim m\gamma L/f_{\mathrm{a}}$, where $L = Nb$ is the rod's length. More explicitly, the (overdamped) equation of motion for the nth monomer of the rod is

$$m\gamma\frac{\partial \mathbf{r}_n(t)}{\partial t} = f_{\mathrm{a}}\hat{\mathbf{p}}(t) + \mathbf{N}(s,t), \tag{3}$$

where $\mathbf{r}_n(t) = \mathbf{R}(t) + nb\hat{\mathbf{p}}(t)$ is the position of the nth monomer. The last term is the normal force due to the boundary. Note that we have assumed the activity-dominated regime, so thermal forces are negligible.

Without loss of generality, we take the boundary to be oriented such that its normal vector is $\hat{\mathbf{y}}$, and the rod to lie in the x-y plane. The normal force can then be written as

$$\mathbf{N}(s,t) = f_{\mathrm{a}}(\hat{\mathbf{p}}\cdot\hat{\mathbf{y}})\left[\left(\frac{\hat{\mathbf{p}}\cdot\hat{\mathbf{y}}}{\hat{\mathbf{p}}\cdot\hat{\mathbf{x}}}\right)\hat{\mathbf{x}} - \hat{\mathbf{y}}\right]\delta_{n,N}. \tag{4}$$

* aparna@brandeis.edu
† hagan@brandeis.edu

Subtracting Eq. (3) for $n = 0$ from that of $n = N$ yields

$$m\gamma L \frac{\partial \hat{\mathbf{p}}}{\partial t} = f_{\mathrm{a}}(\hat{\mathbf{p}} \cdot \hat{\mathbf{y}}) \left[\left(\frac{\hat{\mathbf{p}} \cdot \hat{\mathbf{y}}}{\hat{\mathbf{p}} \cdot \hat{\mathbf{x}}} \right) \hat{\mathbf{x}} - \hat{\mathbf{y}} \right]. \tag{5}$$

The only timescale in this equation is

$$\tau_{\mathrm{rot}} = \frac{m\gamma L}{f_{\mathrm{a}}}. \tag{6}$$

b. Collision timescale: To determine the collision timescale, we first must compute the collisional cross section, σ_{coll}, of two self-propelled rods. Consider two rigid rods of length L and diameter σ. One rod is located at $\mathbf{r}$ with orientation $\hat{\mathbf{p}}$, and the other is located at the origin with orientation $\hat{\mathbf{q}}$. Again neglecting thermal motion, we assume the rods move in the direction of their tangents with an active velocity $v_0 = f_{\mathrm{a}}/m\gamma$. We choose our reference frame such that the rod at the origin is stationary, and the other moves at the relative velocity $\mathbf{v} = v_0(\mathbf{p} - \mathbf{q})$. This defines an axis $\hat{\mathbf{w}} = \mathbf{v}/|\mathbf{v}|$. Projecting the rods onto the plane perpendicular to $\hat{\mathbf{w}}$, we find that both rods have the projected length

$$L' = \left(\frac{1 + \hat{\mathbf{p}} \cdot \hat{\mathbf{q}}}{2} \right) L. \tag{7}$$

We can additionally project the rods' orientations on to this plane:

$$\hat{\mathbf{p}}' = \frac{\hat{\mathbf{p}} - (\hat{\mathbf{p}} \cdot \hat{\mathbf{w}})\hat{\mathbf{w}}}{|\hat{\mathbf{p}} - (\hat{\mathbf{p}} \cdot \hat{\mathbf{w}})\hat{\mathbf{w}}|} = \frac{\hat{\mathbf{p}} + \hat{\mathbf{q}}}{|\hat{\mathbf{p}} + \hat{\mathbf{q}}|} \tag{8}$$

$$\hat{\mathbf{q}}' = \frac{\hat{\mathbf{q}} - (\hat{\mathbf{q}} \cdot \hat{\mathbf{w}})\hat{\mathbf{w}}}{|\hat{\mathbf{q}} - (\hat{\mathbf{q}} \cdot \hat{\mathbf{w}})\hat{\mathbf{w}}|} = \hat{\mathbf{p}}'. \tag{9}$$

Notably the rods are always parallel when projected into this plane. Treating the projected shapes of the rods as simple rectangles of length L' and width σ, the collision region is also a rectangle, with side lengths $2L'$ and 2σ, giving a collision area of

$$A(\hat{\mathbf{p}}, \hat{\mathbf{q}}) = 4L'\sigma = 2L\sigma(1 + \hat{\mathbf{p}} \cdot \hat{\mathbf{q}}). \tag{10}$$

This approximation of the projected rod shape is valid in the long rod limit, $L \gg \sigma$, since accounting for the exact shape of the ends of the rods will only contribute a term of order $\mathcal{O}(\sigma^2)$. Averaging over all orientations $\hat{\mathbf{p}}$ and $\hat{\mathbf{q}}$ leads to the total collisional cross section:

$$\sigma_{\mathrm{coll}} = \int A(\hat{\mathbf{p}}, \hat{\mathbf{q}}) \frac{\mathrm{d}\hat{\mathbf{p}}}{4\pi} \frac{\mathrm{d}\hat{\mathbf{q}}}{4\pi} = \frac{2}{\pi} L\sigma. \tag{11}$$

If the number density of rods is ρ, then the collision timescale is defined by the relation

$$\rho\sigma_{\mathrm{coll}}(L + v_0\tau_{\mathrm{coll}}) = 1. \tag{12}$$

That is, a rod will explore a volume $V(t) = \sigma_{\mathrm{coll}}(L + v_0 t)$ in time t, and therefore will collide with $\rho V(t)$ rods on average in that time. The collision timescale is the time τ_{coll} such that it will have collided with one rod on average. Thus,

$$\tau_{\mathrm{coll}} = \frac{1}{\rho\sigma_{\mathrm{coll}} v_0} - \frac{L}{v_0} \tag{13}$$

c. Competition of timescales: Recall the rotation timescale $\tau_{\mathrm{rot}} = L/v_0$. The collision timescale can be written in terms of τ_{rot} as

$$\tau_{\mathrm{coll}} = \left(\frac{1}{\rho\sigma_{\mathrm{coll}} L} - 1 \right) \tau_{\mathrm{rot}}, \tag{14}$$

We expect that highly-aligned configurations, such as polar rings and caps, will form when $\tau_{\mathrm{coll}} \lesssim \tau_{\mathrm{rot}}$. The number density is related to the volume fraction as $\rho = 4\phi/\pi L\sigma^2$ (treating the rods as cylinders). Thus, we expect a transition provided $2\rho\sigma L \gtrsim 1$, or

$$\phi a \gtrsim (\pi/4)^2, \tag{15}$$

where $a = L/\sigma$ is the rod aspect ratio. Finally, we note that performing the same calculation in 2D results in the same scaling.

3. Calculation of number of caps

a. Derivation of the effective 'free energy': We assume that activity induces an effective attractive interaction between active rods, leading rods to preferentially form smectic layers. These smectic layers are sheared by the curvature of the confining vesicle. We assume that there is a force that is linear in displacement that resists this shear, as well as an interfacial tension at the edges of the cap. Using the parameterization shown in Fig. 2b of the main text, we align the cap to the z-axis and write the height field of the cap as a function of the polar angle θ:

$$h(\theta) = R_{\text{ves}} \cos\theta. \tag{16}$$

Note that we have assumed the cap radius is the same as the undeformed vesicle radius. Active forces may deform the vesicle at the caps, leading to caps having a radius that is different from the vesicle's unperturbed radius. Thus, this calculation will be most accurate when the vesicle is not deformed too much from its initial state (high vesicle stiffness or low activity).

The gradient in the height field gives the local shear energy density, which can be integrated over the cap surface to find the total shear energy, with shear modulus $G \sim f_{\text{a}}$:

$$\begin{aligned} u_{\text{shear}}(\theta) &= \frac{1}{2} G \int \mathrm{d}S \left| \frac{\partial h}{\partial (R_{\text{ves}} \sin\theta)} \right|^2 \\ &= \pi R_{\text{ves}}^2 G \int_0^\theta \mathrm{d}\theta' \sin\theta' \tan^2\theta' \\ &= \pi R_{\text{ves}}^2 G \frac{(1-\cos\theta)^2}{\cos\theta}. \end{aligned} \tag{17}$$

The cap has radius $R_{\text{ves}} \sin\theta$, and therefore the interfacial energy can be written as

$$u_{\text{int}}(\theta) = 2\pi R_{\text{ves}} \lambda \sin\theta, \tag{18}$$

where $\lambda \sim f_{\text{a}} L$ is the interfacial tension.

If the total number of rods in the system is N_{fil}, and we assume that all caps are the same size, then the total number of caps is given by

$$n_{\text{cap}} = \frac{N_{\text{fil}}}{2\pi R_{\text{ves}}^2 \rho (1-\cos\theta)}, \tag{19}$$

where $\rho = 2/\sqrt{3}\sigma^2$ is the hexagonal close packing density, and σ is the rod diameter. Therefore, the total energy (relative to a reference state corresponding to a perfectly aligned smectic layer of rods) is

$$\begin{aligned} U(\theta) &= n_{\text{cap}}(\theta)[u_{\text{shear}}(\theta) + u_{\text{int}}(\theta)] \\ &= \frac{G N_{\text{fil}}}{\rho(1-\cos\theta)} \left[\frac{(1-\cos\theta)^2}{2\cos\theta} + \frac{\lambda}{G R_{\text{ves}}} \sin\theta \right]. \end{aligned} \tag{20}$$

From this, we define the (normalized) free energy per filament $f(\theta)$ as

$$f(\theta) = \frac{\rho U(\theta)}{G N_{\text{fil}}} = \frac{1}{1-\cos\theta} \left[\frac{(1-\cos\theta)^2}{2\cos\theta} + \zeta \sin\theta \right], \tag{21}$$

where $\zeta = \lambda / G R_{\text{ves}} = cL/R_{\text{ves}}$, with the proportionality constant c the only free parameter.

b. Computing the number of caps: Using Eq. (21), we first compute the optimal θ as

$$\theta^* = \arg\min_\theta f(\theta) \tag{22}$$

and then evaluate $n_{\text{cap}}(\theta^*)$. This yields the number of caps as a function of the parameter ζ, which we can fit to our simulation data.

Provided ζ is small, we can find an asymptotic solution in the following way. Let $z = \cos\theta/(1-\cos\theta)$ for convenience. Since $z(\theta)$ is a monotonic function of θ (for $0 \leq \theta < \pi$), minimizing Eq. (21) with respect to z is equivalent to minimizing with respect to θ. The free energy can then be written as

$$f(z) = \frac{1}{2z} + \zeta\sqrt{1+2z}. \tag{23}$$

Taking the derivative and setting it to zero, we obtain the following equation for z:

$$\frac{\zeta}{\sqrt{1+2z}} - \frac{1}{2z^2} = 0. \tag{24}$$

Squaring both sides, we can rearrange this into the form

$$\frac{z^4}{1+2z} = \frac{1}{4\zeta^2}. \tag{25}$$

If $\zeta \ll 1$, then the right hand side is very large and so we can assume that $z \gg 1$ as well. Approximating $1+2z \approx 2z$, this reduces to

$$z \approx (2\zeta^2)^{-1/3} \tag{26}$$

or, equivalently,

$$\cos\theta \approx \frac{1}{1+(2\zeta^2)^{1/3}}. \tag{27}$$

Recall that we expect $\zeta \propto L/R_{\mathrm{ves}}$. We can instead write ζ as a ratio of the rod aspect ratio a to a critical aspect ratio, $\zeta = a/a^*$, with $a^* \cong R_{\mathrm{ves}}/\sigma \gg 1$. Inserting Eq. (27) into Eq. (19), and absorbing constants into a^*, we obtain the result shown in the main text

$$n_{\mathrm{cap}} = AN_{\mathrm{fil}}[1+(a/a^*)^{-2/3}], \tag{28}$$

where $A = 1/2\pi\rho R_{\mathrm{ves}}^2$.

Dependence of a^* on activity and bending modulus: We anticipate that the critical aspect ratio may depend on activity for several reasons. First, the critical aspect ratio will depend on the *local* vesicle curvature in the vicinity of the cap, $a^* \cong R_{\mathrm{ves}}^{\mathrm{local}}/\sigma$, with $R_{\mathrm{ves}}^{\mathrm{local}} \leq R_{\mathrm{ves}}$ because the active force from the rods will locally deform the vesicle from its unperturbed curvature. The extent of deformation will increase with increasing activity and decreasing vesicle bending modulus. For simplicity, we have assumed $R_{\mathrm{ves}}^{\mathrm{local}} = R_{\mathrm{ves}}$ in the preceding analysis. Second, at very low activity $f_{\mathrm{a}} \lesssim k_{\mathrm{B}}T/\sigma$ (or $f_{\mathrm{a}} \lesssim 1$ in our units), thermal motions become relevant and the caps are not as well-formed (see Supplementary Figure 2 and SI Movie 4).

4. Onset of dynamic caps

Assuming all N_{fil} rods are contained within a cap, the total area occupied by rods is

$$A_{\mathrm{tail}} = N_{\mathrm{fil}}/\rho, \tag{29}$$

where, as before, $\rho = 2/\sqrt{3}\sigma^2$ is the hexagonal close packing density. Now, let us consider a state with all rods organized into caps, and assume that the vesicle is approximately spherical with radius R_{ves}. Then, the tails of the rods will be located approximately on the surface of an inner spherical region, with radius $R = R_{\mathrm{ves}} - L_{\mathrm{rod}}$. If the total area of rod tails A_{tail} occludes a large fraction of the surface area of this inner region, then the tails of the rods are likely to interact. Such interactions can disrupt the stability of the caps, potentially leading to motions and/or breakup of caps. This occurs when

$$N_{\mathrm{fil}}/\rho \gtrsim 4\pi(R_{\mathrm{ves}} - L_{\mathrm{rod}})^2 \rightarrow N_{\mathrm{fil}} \gtrsim 4\pi\rho R_{\mathrm{ves}}^2(1 - L_{\mathrm{rod}}/R_{\mathrm{ves}})^2. \tag{30}$$

Based on this analysis, caps will become motile above threshold values of the length and number of filaments given by Eq. (30).

We can recast these results in terms of the volume fraction ϕ and aspect ratio a of the filament. Using $N_{\mathrm{fil}} \sim \phi/a$, we write

$$\phi \gtrsim Ca(1 - a\sigma/R_{\mathrm{ves}})^2, \tag{31}$$

where $C \propto L_{\mathrm{rod}}/R_{\mathrm{ves}}$. Consistent with this result, our simulation results show that motile caps arise in the lower-right region of the parameter space of Fig. 1 of the main text.

5. Effects of filament semiflexibility

As we show in Sec. A, the filament orientation correlation timescale is

$$\tau_{\rm corr} = m\gamma\sigma^2 a^3/12k_{\rm B}T. \tag{32}$$

We assume that for semiflexible rods we can make the substitution $a \to l_{\rm p}/\sigma$, where $l_{\rm p}$ is the filament persistence length. Further, simulations show [4]

$$l_{\rm p} = \frac{2\kappa b/k_{\rm B}T}{1+(f_{\rm a}\sigma/k_{\rm B}T)^2} = \frac{2b\chi_{\rm fil}}{1+{\rm Pe}^2}, \tag{33}$$

where $b = \sigma/2$ is the bond length between filament monomers and κ is strength of the harmonic angle potential that controls the semiflexibility of the filament. Assuming that Pe is large, we find

$$l_{\rm p}/\sigma \approx \frac{\chi_{\rm fil}}{{\rm Pe}^2}. \tag{34}$$

The system enters the strong confinement limit when $\tau_{\rm corr} \gtrsim 2R_{\rm ves}/v_0$, where $v_0 = f_{\rm a}/m\gamma$ is the filament propulsion velocity. Using Eq. (34), this can be rearranged to find

$$ {\rm Pe}^{\rm SC} \gtrsim C\chi_{\rm fil}^{3/5}, \tag{35}$$

where $C = (\sigma/24R_{\rm ves})^{1/5}$. This is shown as a dashed line in Fig. 5a of the main text.

Note that there are no fitting parameters used in this analysis.

6. Vesicle deformation in the polar ring state

In the polar ring state, the deformation of the vesicle leads to a restoring force that is balanced by the internal active stresses generated by the filaments. Assuming the shape of the vesicle is an oblate ellipsoid with minor radius R_1 and major radius R_2, we can parameterize the vesicle surface as

$$\mathbf{r}(\theta,\phi) = R_1\cos\phi(\cos\theta\hat{\mathbf{x}} + \sin\theta\hat{\mathbf{y}}) + R_2\sin\phi\hat{\mathbf{z}}, \tag{36}$$

with $0 \le \theta < 2\pi$ and $0 \le \phi < \pi$. Using this parameterization, the mean curvature can be written as

$$H(\theta,\phi) = -\sqrt{2}\frac{R_1}{R_2}\frac{R_1^2+R_2^2+(R_2^2-R_1^2)\cos^2\phi}{(R_1^2+R_2^2+(R_2^2-R_1^2)\cos 2\phi)^{3/2}} \tag{37}$$

In the following, we assume that $0 < R_1 \le R_0 \le R_2$, and that the total vesicle area $A = 4\pi R_0^2$ is conserved. This latter assumption allows us to write both R_1 and R_2 in terms of a single parameter ν, defined as $\cosh\nu = R_2/R_1$, that describes the vesicle deformation:

$$R_1 = R_0\sqrt{\frac{4\tanh\nu}{2\nu+\sinh 2\nu}} \quad \text{and} \quad R_2 = R_0\sqrt{\frac{2\sinh 2\nu}{2\nu+\sinh 2\nu}}. \tag{38}$$

We can then explicitly compute the deformation energy as

$$U = \frac{1}{2}\kappa\int {\rm d}A\,(2H)^2 = \frac{2}{3}\pi\kappa\left(8+\cosh 2\nu + \frac{6\nu}{\sinh 2\nu}\right). \tag{39}$$

In the small deformation limit, $\nu \to 0$, we can approximate the inverse of Eq. (38) as

$$\nu \approx \sqrt{6\delta}, \tag{40}$$

where $\delta = (R_2 - R_0)/R_0$ is a measure of the deformation of the vesicle relative to the undeformed radius. Using this we can write the restoring force to linear order in the relative deformation as

$$-f_{\rm R} = \frac{dU}{dR_2} \approx \left(\frac{384\pi\kappa}{5R_0}\right)\delta + O(\delta^2). \tag{41}$$

We can now turn to computing the active forces that balance the restoring force due to vesicle deformation. Let

$$\mathbf{r}_n(t) = R_2[\cos(\omega t - \phi_n)\hat{\mathbf{x}} + \sin(\omega t - \phi_n)\hat{\mathbf{y}}] = R_2\hat{\mathbf{r}}_n(t) \quad (42)$$

be the position of the *head* of the nth rigid active rod, with ω and angular velocity and ϕ_n the phase angle setting the initial position of the rod on the vesicle surface. The orientation of the rod is $\hat{\mathbf{p}}_n(t)$. Requiring the head and tail to both make contact with the vesicle leads to the condition

$$|\mathbf{r}_n(t)| = |\mathbf{r}_n(t) - L\hat{\mathbf{p}}_n(t)| \implies \hat{\mathbf{r}}_n \cdot \hat{\mathbf{p}}_n = \frac{L}{2R_2}, \quad (43)$$

with $L = a\sigma$ the length of each rod. Since each rod must be moving in circular motion, we require the radial component of the force on each rod to be zero. Thus, we can see that

$$f_{\mathrm{R}} = \sum_n f_{\mathrm{a}}\hat{\mathbf{p}}_n \cdot \hat{\mathbf{r}}_n = \frac{N_{\mathrm{fil}} L \xi f_{\mathrm{a}}}{2R_2} \quad (44)$$

where $\xi f_{\mathrm{a}} \frac{L}{2R_2}$ is the total normal force exerted by a filament on the vesicle. We expect $1 < \xi < M$, since since filament monomers near the filament head will contribute a normal force $\sim f_{\mathrm{a}} L/2R_2$ whereas those near the filament center will not exert significant normal forces.

Finally, equating Eq. (41) and Eq. (44) yields

$$\delta \approx \frac{1}{2}\left(\sqrt{1 + C a N_{\mathrm{fil}} \mathrm{Pe}} - 1\right), \quad (45)$$

where the constant $C = 5k_{\mathrm{B}}T\xi/96\sqrt{3}\pi\kappa_{\mathrm{ves}}$ (where we have used $\kappa = \sqrt{3}\kappa_{\mathrm{ves}}/2$ for the continuum bending modulus). For $\kappa_{\mathrm{ves}} = 5000k_{\mathrm{B}}T$ as used primarily in the simulations in the main text, we find $C \approx 1.914 \times 10^{-6}\xi$. As we show in Supplemental Figure 6, the vesicle deformations indeed follow the form of Eq. (45), with a fit value of $C \approx 2.3 \times 10^{-5}$. This would imply $\xi \approx 12$ which is reasonable given the argument above and the range of rod lengths used in Supplemental Figure 6, $M = 40, 50$. However, we also note that we have assumed a constant vesicle area and small deformation limit in this calculation, whereas in the simulations the area is not explicitly constrained and in many cases the vesicle deformations exceed the small deformation limit.

SUPPLEMENTARY METHODS

1. Equations of motion and simulation details

We simulate a vesicle with $N_{\mathrm{ves}} = 2432$ monomers and a nominal radius of $R_{\mathrm{ves}} \approx 25\sigma$, measured as the distance from the center of mass of the vesicle to the center of any given monomer within the vesicle in its undeformed state. For this radius, we set the vesicle monomer diameter to $\sigma_{\mathrm{ves}} = a\sigma$ with $a \approx 1.934$ to ensure that there were no holes in the vesicle that the active filaments could escape from. A filament is a linear chain of M monomers, each with diameter σ. The vesicle is filled with N_{fil} such filaments, with N_{fil} varied to control the volume fraction $\phi = N_{\mathrm{fil}}V_{\mathrm{fil}}/V_{\mathrm{ves}}$. See Supplementary Figure 3.

Bonded monomers in both the vesicle and the filaments interact through an expanded FENE potential:

$$U_{\mathrm{FENE}}(r) = -\frac{1}{2}k_{\mathrm{stretch}}\Delta^2 \ln\left[1 - \left(\frac{r-b}{\Delta}\right)^2\right], \quad (46)$$

where r is the distance between the two bonded monomers, k_{stretch} is the bond strength, b is the preferred bond length, and Δ is the maximum deviation; that is, $|r - b| < \Delta$. For the vesicle, we set $k_{\mathrm{ves}} = 1000k_{\mathrm{B}}T/\sigma^2$, $b_{\mathrm{ves}} \approx 1.934\sigma$ (for hexamer bonds) or $b_{\mathrm{pent}} \approx 1.645\sigma$ (for pentamer bonds), and $\Delta = b/2$. For the active filaments, we set $k_{\mathrm{fil}} = 2000k_{\mathrm{B}}T/\sigma^2$, $b_{\mathrm{fil}} = 0.5\sigma$, and $\Delta = 0.4\sigma$. These stiff bond potentials act to nearly constrain the length and area of the filaments and vesicle, respectively.

To penalize curvature, neighboring triangles on the vesicle (those that share an edge) also interact through a harmonic dihedral potential. Each triangle i defines a unique normal vector $\hat{\mathbf{n}}_i$. For neighboring triangles i and j, the interaction potential is given by

$$U_{\mathrm{dih}}(\phi) = \kappa_{\mathrm{dih}}(1 - \cos\phi), \quad (47)$$

where $\cos\phi = \hat{\mathbf{n}}_i \cdot \hat{\mathbf{n}}_j$. For most simulations in the main text, we set $\kappa_{\text{ves}} = 5000 k_{\text{B}}T$ so that the vesicle has a relatively large bending modulus $\kappa = \sqrt{3}\kappa_{\text{ves}}/2$ and Young's modulus $Y = 2k_{\text{ves}}/\sqrt{3}$ [5–7]. Infinitely rigid vesicles are implemented by not integrating the equations of motion for the vesicle monomers so that they are always in their initial (spherical) configuration. Note that for vesicle rigidities such that faceting occurs (FvK $\gtrsim 154$ [8]), the facets can act as nucleation points for cap formation, making caps more likely to form rather than, for example, ring states, as is seen in Fig. 6a.

Filament semiflexibility is incorporated through a harmonic angle potential of the form:

$$U_{\text{angle}}(\theta) = \kappa_{\text{fil}}\theta^2, \tag{48}$$

where θ is the angle between two neighboring bonds i and j; that is, $\cos\theta = \hat{\mathbf{b}}_i \cdot \hat{\mathbf{b}}_j$, where $\hat{\mathbf{b}}_{\{i,j\}}$ are neighboring bond vectors.

Non-bonded monomers interact sterically through an expanded WCA potential given by

$$U_{\text{WCA}}(r) = 4\epsilon\left[\left(\frac{\sigma}{r-\Delta}\right)^{12} - \left(\frac{\sigma}{r-\Delta}\right)^{6}\right] \quad r < r_c, \tag{49}$$

where r is the distance between two interacting monomers, ϵ is the strength of the interaction, Δ shifts the potential, and $r_{\text{cut}} = 2^{1/6}\sigma + \Delta$ is a cutoff distance. For interactions between two filament monomers, we set $\Delta = 0$, and for interactions between a filament monomer and a vesicle monomer we set $\Delta = \sigma_{\text{ves}}/2$. This ensures that active filaments cannot escape the vesicle. In all cases, we set $\epsilon = k_{\text{B}}T$. We do not consider excluded volume interactions between two vesicle monomers as the stiff bond and dihedral potentials act to keep vesicle monomers far apart.

Given these interactions, the total energy is the sum

$$\begin{aligned} U_{\text{tot}} = &\sum_{\text{pairs}} U_{\text{WCA}}(r) + \sum_{\text{bonds}} U_{\text{FENE}}(r) \\ &+ \sum_{\text{angles}} U_{\text{angle}}(\theta) + \sum_{\text{dihedrals}} U_{\text{dih}}(\phi). \end{aligned} \tag{50}$$

The equation of motion for any given atom i within molecule α is then given by

$$\begin{aligned} m\frac{\partial^2 \mathbf{r}_i^\alpha}{\partial t^2} = &-\frac{\partial U_{\text{total}}}{\partial \mathbf{r}_i^\alpha} - m\gamma\frac{\partial \mathbf{r}_i^\alpha}{\partial t} \\ &+ (1-\delta_{\alpha,0}) f_{\text{a}} \frac{\mathbf{r}_{i+1}^\alpha - \mathbf{r}_{i-1}^\alpha}{|\mathbf{r}_{i+1}^\alpha - \mathbf{r}_{i-1}^\alpha|} + \boldsymbol{\xi}_i^\alpha(t). \end{aligned} \tag{51}$$

Here, $\alpha = 0$ represents the vesicle, while $\alpha > 0$ represents a filament. The first term on the right hand side consists of the conservative forces present in the system, the second term damps the motion through a viscous force (with the damping coefficient set to $\gamma = 1/\tau$), the third term applies an active force to the atoms of each filament along the tangent, and the last term adds random thermal forces. The thermal forces are modeled as Gaussian white noise with moments

$$\begin{aligned} \langle \boldsymbol{\xi}_i^\alpha(t) \rangle &= 0, \quad \text{and} \\ \left\langle \boldsymbol{\xi}_i^\alpha(t) \cdot \boldsymbol{\xi}_j^\beta(t') \right\rangle &= 6\gamma k_{\text{B}}T \delta_{ij}\delta_{\alpha\beta}\delta(t-t'). \end{aligned} \tag{52}$$

Simulations are run with a timestep of $\delta t = 10^{-3}\tau$ for a total time of $10^4\tau$. For the two state diagrams shown in the main text (Fig. 1 and Fig. 3), we ran 9 trials for each set of parameters. For the state diagram shown in Fig. 5 of the main text we only ran one trial. The first 10% of the simulation is used for initialization, during which the filament positions and orientations are randomized. The final 10% of the simulation is used to analyze the resulting vesicle conformations and filament organizations. Note that the system reaches a long-lived steady state configuration very rapidly, and tends to exist in that state for nearly the entirety of the simulations (see Supplementary Figure 4).

2. State detection algorithm

We classify states by a combination of the vesicle conformation and the organization of the rods within the vesicle. The classifications are as follows

- *undeformed/spherical*: the vesicle is in a nearly spherical state (symbolized by $\bullet$)
- *deformed/other*: the vesicle is in a deformed state, but the rod organization is not consistent or difficult to detect; typically, this occurs for states in which the filaments form unstable polar rings that form and break apart over time (symbolized by $\star$)
- *oblate/ring*: the vesicle is an oblate spheroid, and the filaments are organized into a stable polar ring (symbolized by $\circ$)
- *capped*: the filaments are organized into stable caps, with varying amounts of vesicle deformation (symbolized by $\circ$ with the number of intersecting lines in the symbol equal to the median number of detected caps)
- *dynamic capped*: the filaments are organized into caps that are dynamic and/or motile (symbolized by $\diamond$)

Our classification algorithm is constructed as follows. We first classify the vesicle conformation. We define the asphericity α of the vesicle to be

$$\alpha = \frac{1}{6\sqrt{\pi}} \frac{S^{3/2}}{V}, \tag{53}$$

where S is the surface area of the vesicle and V is the volume. Note that $\alpha = 1$ for a sphere, and $\alpha > 1$ for any non-spherical shape. Additionally, we measure the gyration tensor G as

$$G = \frac{1}{N_{\text{ves}}} \sum_{i=1}^{N_{\text{ves}}} \vec{r}_i \otimes \vec{r}_i, \tag{54}$$

where N_{ves} is the number of monomers making up the vesicle, and $\vec{r}_i$ is the position of the ith monomer. Let a, b, and c be the eigenvalues of G such that $a \leq b \leq c$. Then define

$$x = (b-a)/(b+a)$$
$$y = (c-b)/(c+b).$$

Using α, x, and y, we classify the vesicle conformation into four categories:

- *spherical*: $\alpha \leq 1.01$ and $x \leq 0.1$ and $y \leq 0.1$,
- *oblate*: $x > 0.1$ and $y \leq 0.1$,
- *prolate*: $x \leq 0.1$ and $y > 0.1$,
- *non-spherical*: otherwise.

Next, we attempt to classify the filament organization.

First, we determine if there are any caps in the system. To do this, we compute two fields on the vesicle surface: a polarization field $\mathbf{p}_i$ and an order parameter q_i that measures how perpendicular the filaments are to the vesicle, both of which are evaluated at each vesicle monomer i. Let $\hat{\mathbf{n}}_i$ be the unit normal vector of the vesicle at the ith vesicle monomer, and let M_i be the set of filament monomers that are within 4σ of the ith vesicle monomer. Then

$$\mathbf{p}_i = \frac{\sum_{j \in M_i} \hat{\mathbf{t}}_j}{\left|\sum_{j \in M_i} \hat{\mathbf{t}}_j\right|}, \tag{55}$$

where $\hat{\mathbf{t}}_j$ is the unit tangent vector of the filament evaluated at filament monomer j. Similarly,

$$q_i = \frac{1}{|M_i|} \sum_{j \in M_i} \hat{\mathbf{t}}_j \cdot \hat{\mathbf{n}}_i \tag{56}$$

To find potential caps, we first find the vesicle monomer i^* such that q_{i^*} is maximized. We then use the procedure described in Algorithm 1 to find a cap based on this seed monomer. To find additional caps, we choose a new seed point by finding another monomer j^* such that q_{j^*} is the maximal value among all monomers that are not yet a member of a cap. We continue this process until all vesicle monomers have been visited, or until we do not find any additional caps. Once all candidate caps are found, we discard any caps that comprise fewer than $\lceil \sqrt{N_{\text{ves}}} \rceil = 50$ monomers.

Finally, as done for the vesicle itself, we compute the smallest and largest eigenvalues, λ_{min} and λ_{max} respectively, of the gyration tensor computed using only the filament monomers. Let $z = 1 - \lambda_{\text{min}}/\lambda_{\text{max}}$. Then we classify the filament organization as one of the following:

```
input  : A seed vesicle monomer i*
output : A list of vesicle monomers belonging to a cap
initialize cap to empty list;
initialize visited to empty set;
initialize todo to stack containing only i*;
while todo is not empty do
    i ← value popped from todo;
    insert i into visited;
    if p̂_i · p̂_{i*} ≥ cos(π/6) then
        append i to cap;
        N_i ← set of monomers neighboring monomer i;
        for j ∈ N_i do
            if j not in visited and p̂_j · p̂_{i*} ≥ cos(π/6) then
                push j on to todo;
            end
        end
    end
end
```

Algorithm 1: Algorithm to determine vesicle monomers belonging to a cap

- *capped*: if there are a non-zero number of caps,
- *polar ring*: if there are no caps, and $z > 0.75$,
- *other*: otherwise.

The class *other* is very broad, and can include a range of filament organizations and behaviors such as polar flocks on the vesicle surface, or transient polar rings that continuously form and break apart. Usually these states do not result in any large scale deformation of the vesicle, but in some cases the transient ring states can lead to measurable deformation.

For each simulation, we take 11 samples from the last 10% of the trajectory and measure the vesicle conformation and filament organization at each sample. If we also have multiple trials, then we aggregate these identifications. Finally, we assign an conformation and organization based on the most common measurement.

Having information on both the vesicle conformation and filament organization, we can revisit our earlier classification scheme:

- *undeformed/spherical*: the filament organization is *other*, and the vesicle conformation is *spherical*,
- *deformed/other*: the filament organization is *other*, and the vesicle conformation is not *spherical*,
- *oblate/ring*: the filament organization is *polar ring*, and the vesicle conformation is *oblate*,
- *capped*: the filament organization is *capped*,
- *dynamic capped*: the filament organization is *capped*, but the number of caps is inconsistent.

For the dynamic capping states, we classify the number of caps as inconsistent if the majority of states are capped, but no particular number of caps forms a majority of these states.

SUPPLEMENTARY REFERENCES

[1] Fily, Y., Baskaran, A. & Hagan, M. F. Dynamics of self-propelled particles under strong confinement. Soft Matter **10**, 5609–5617 (2014).

[2] Fily, Y., Baskaran, A. & Hagan, M. F. Active particles on curved surfaces (2016). 1601.00324.

[3] Doi, M. & Edwards, S. F. The Theory of Polymer Dynamics. No. 73 in International Series of Monographs on Physics (Clarendon Press, Oxford, 2007).

[4] Joshi, A., Putzig, E., Baskaran, A. & Hagan, M. F. The interplay between activity and filament flexibility determines the emergent properties of active nematics. Soft Matter **15**, 94–101 (2019).

[5] Seung, H. S. & Nelson, D. R. Defects in flexible membranes with crystalline order. Phys. Rev. A **38**, 1005–1018 (1988).

[6] Gompper, G. & Kroll, D. M. Random Surface Discretizations and the Renormalization of the Bending Rigidity. J. Phys. I France **6**, 1305–1320 (1996).

[7] Lázaro, G. R., Dragnea, B. & Hagan, M. F. Self-assembly of convex particles on spherocylindrical surfaces. Soft Matter **14**, 5728–5740 (2018).
[8] Lidmar, J., Mirny, L. & Nelson, D. R. Virus shapes and buckling transitions in spherical shells. Phys. Rev. E **68**, 051910 (2003).

SUPPLEMENTARY FIGURES

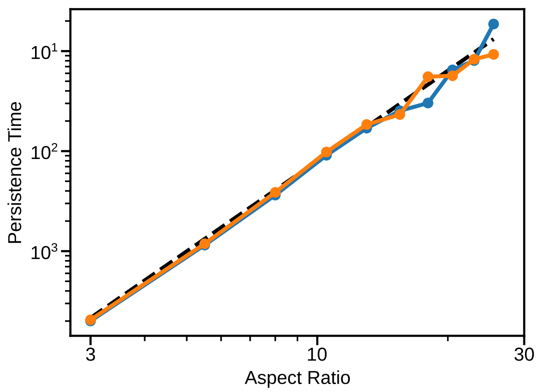

FIG. 1. Filament rotational diffusion time is independent of activity in the absence of filament-filament collisions. The filament orientation persistence time scales with aspect ratio as $\tau_{\mathrm{corr}} \propto a^3$ in the rigid rod limit ($\chi_{\mathrm{fil}} = 10^4$). The blue dots and orange dots are data from simulations with the active force turned off and on, respectively, showing that activity does not affect the persistence time. The dashed line follows $\tau_{\mathrm{corr}} = m\gamma\sigma^2 a^3/12k_{\mathrm{B}}T$.

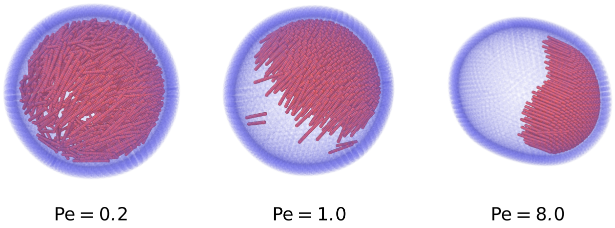

FIG. 2. Caps that form at lower active force strengths tend to be more ragged and drive less vesicle deformation than those that form at larger active force strengths. Snapshots are taken from trials used in Fig. 3 for $\phi = 0.1$.

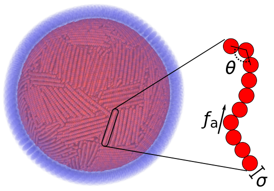

FIG. 3. Example schematic of a system of confined active filaments. The vesicle is constructed from a triangulated mesh of N_{ves} beads, and contains N_{fil} polar active filaments. Each filament consists of M bonded monomers of diameter σ. A propulsion force with magnitude f_{a} is applied to each monomer in the direction of the local filament tangent, and the filament bending modulus is κ_{fil}.

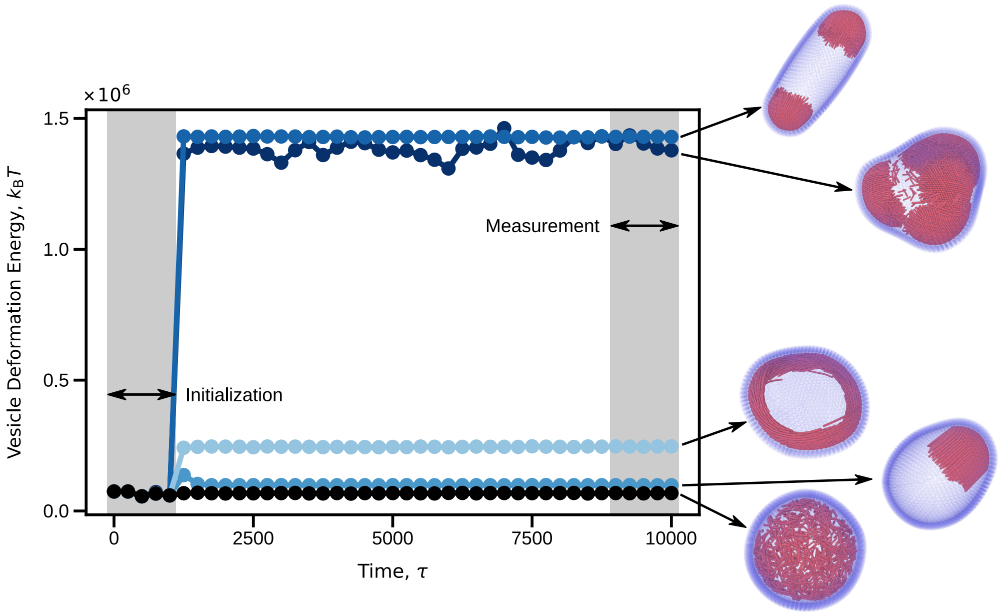

FIG. 4. Measurements of the total deformation energy of the vesicle show that the systems rapidly enter a dynamical steady state. Systems with many motile caps (dark blue) show transient energy fluctuations over all time, while all other simulations plateau at a fixed deformation energy. The first 10% of the simulation (left shaded region) is the initialization phase, during which the filaments' positions and orientations are effectively randomized. We perform all measurements during the final 10% of the simulation (right shaded region), which is well beyond the point at which systems have reached a dynamical steady state.

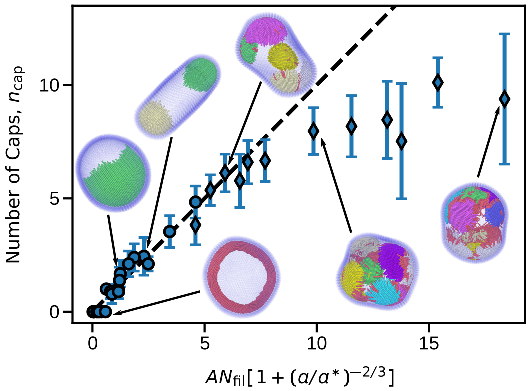

FIG. 5. Number of caps formed in simulations compared to theory, viewed on a linear scale. This is the same data as shown in Fig. 4 in the main text. The linear scale allows the ring states with no caps to be shown along with the cap-forming states.

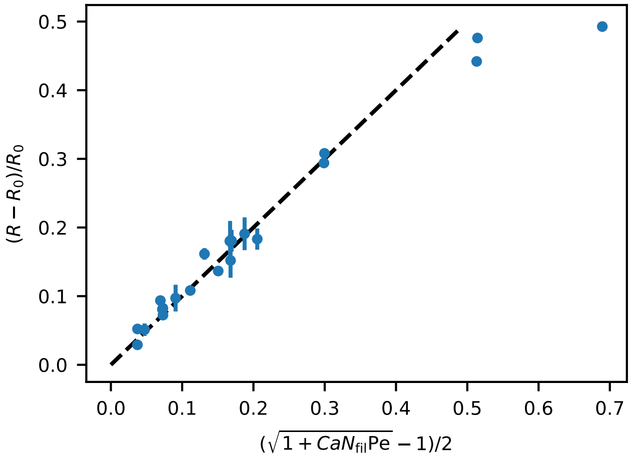

FIG. 6. Vesicle deformation as a function of simulation parameters compared the theoretical estimate. The vesicle deformation, measured relative to the undeformed radius, scales as expected from Eq. (45), with a best fit value of $C \approx 2.3 \times 10^{-5}$ (dashed line). The symbols correspond to the mean relative deformation for ring-forming states taken from Fig. 1 and Fig. 3 from the main text, averaged across trials. Error bars are shown if multiple trials for a given set of parameters form a polar ring, and represent the standard deviation of the relative deformation.
