# Supplementary material for: Vesicle shape transformations driven by confined active filaments
Source: Nat Commun. 2021 Dec 13;12:7247. doi: 10.1038/s41467-021-27310-8 (PMC8668962; doi:10.1038/s41467-021-27310-8)
Supplement: Supplementary file 3 — Description of additional Supplementary File [file 41467_2021_27310_MOESM3_ESM.pdf]

Description for additional supplementary data files

Supplementary movie-1: Animations corresponding to the data shown in Fig. 1. Each element of the figure corresponds to an independent simulation performed at the indicated parameter values, with the volume fraction ϕ increasing along rows, and the aspect ratio a increasing along the columns. Other parameters are $\mathrm{Pe} = 8$ and $\chi_{\mathrm{fil}} = 10^4$.

Supplementary movie-2: Animations corresponding to the data shown in Fig. 3, with the volume fraction ϕ increasing along rows, and the active force strength Pe increasing along the columns. Other parameters are $a = 10.5$ and $\chi_{\mathrm{fil}} = 10^4$.

Supplementary movie-3: Animations corresponding to the data shown in Fig. S2, with the filament stiffness χ_{fil} increasing along rows, and the active force strength Pe increasing along the columns. Other parameters are $\phi = 0.20$ and $a = 10.5$.

Supplementary movie-4: Animations of simulations performed at $\mathrm{Pe} \in \{1, 2, 3\}$ for $\phi = 0.2$, $a = 10.5$, and $\chi_{\mathrm{fil}} = 10^4$. These videos show that decreasing the activity leads to smaller vesicle deformations, more ragged caps, and more frequent rod dissociation from caps.

Supplementary movie-5: Animations of simulations performed at $\phi \in [0.01, 0.10]$ and $\chi_{\mathrm{ves}} \in [10^2, 10^4]$ for $\mathrm{Pe} = 8$, $\phi = 0.1$, $a = 10.5$, and $\chi_{\mathrm{fil}} = 10^4$. These videos show the faceting transition of the vesicle as the rigidity is reduced. Caps are still readily formed and stable, but the polar rings tend to trace a path between facets which destabilizes them.

Supplementary movie-6: Animation of a simulation performed at $\mathrm{Pe} = 8$, $\chi_{\mathrm{fil}} = 10^4$, $\phi = 0.05$ and $a = 25.5$ in an infinitely rigid vesicle. This video shows that the polar rings that form within a rigid vesicle are unstable, and will break apart and reform repeatedly. In contrast, these parameters lead to a stable polar ring when the vesicle has finite rigidity.
